# The Power of Affection: Exploring the Key Drivers of Customer Loyalty in Virtual Reality-Enabled Services

**DOI:** 10.3389/fpsyg.2022.850896

**Published:** 2022-04-25

**Authors:** Jun Yan, Ihtesham Ali, Rizwan Ali, Yaping Chang

**Affiliations:** ^1^School of Management, Huazhong University of Science and Technology, Wuhan, China; ^2^Department of Management Sciences, Lahore Garrison University, Lahore, Pakistan

**Keywords:** virtual reality-enabled services, TAM, authenticity, affective response, customer loyalty

## Abstract

The accelerating growth of virtual reality (VR) technology and evolving customer needs make multifarious challenges and opportunities for service industries. Based on the Technology Acceptance Model (TAM) and Theory of Affection Responses, we explored the key drivers of customer loyalty in virtual reality-enabled services through a large-scaled survey data collected from VR users in four major cities of Pakistan. The study employs the partial least squares structural equation modeling (PLS-SEM). We verified that the authenticity of the VR experience and TAM dimensions (ease of use, usefulness of VR) are the key drivers of customer loyalty béhavioral in VR-enabled services. Furthermore, results revealed that Affective responses (i.e., enjoyment, emotional involvement, and flow state) significantly mediated the relationships between the drivers and customer loyalty (continued use, recommendation, and willingness to pay premium). Implications for researchers and VR practitioners were also provided.

## Introduction

Virtual reality (VR) is reshaping the way how companies and customers interact inventively (Orús et al., 2021). In VR, users are engaged and interact in a computer-generated environment, triggering real-time simulation of users’ senses, further inducing an immersive experience (Guttentag, 2010; Flavián et al., 2019a). Wedel et al. (2020) pointed out that the current global VR application market size is approximately US$ 14 billion and will increase up to US$ 50–60 billion in the next 5 years. Furthermore, the Statista (2018) Report projects that the global market size for VR and augmented reality (AR) will grow from $ 27 billion in 2018 to $ 209.2 billion in 2022.

Virtual reality-enabled services have great potential for service industries to generate unprecedented real-virtual experience. Berger et al. (2007) revealed that VR-enabled services provide realistic experiences through immersive environments similar to people’s real feelings; as a result, potential customers experience and collect information in 3D. Experts have predicted that VR services are changing the individual’s virtual experience (Flavián et al., 2019a) and will be promoted rapidly in several sectors, including tourism (Huang et al., 2016; Yung and Khoo-Lattimore, 2019), online retailing (Bonetti et al., 2018), and marketing (Loureiro et al., 2019). For example, Navitaire, an Amadeus global travel and technology company, introduced immersive VR-enabled travel booking services to enhance travelers’ trip purchase experience (Vallantin, 2017); users can spin a virtual globe to explore, compare destinations, and read the aircraft seat map to select seats. Similarly, Swedish furniture retailer, Ikea, established a VR-equipped showroom on their website. Shoppers can check various fabrics, change the colors of walls, and try multiple combinations in multiple settings (Demodern., 2020). Walmart, a giant retailer, adopted a VR assessment tool to evaluate employees’ potentials by creating computer-simulated in-store environments to assess the knowledge of the employees. For example, a VR in-store simulation situation enables employees to find themselves in busy aisles, confronting various problems, such as spillage, scattered goods, and trash; a manager has 30 s to sort out which problem to fix first (Holley, 2019).

Davis (1989) has developed the Technology Acceptance Model (TAM), which describes the individuals’ acceptance of new technology. TAM has two determines which are considered the two major factors of an individual’s IT acceptance and behavioral intention toward using new technology. Perceived usefulness refers to “the extent that people believe information technology will help them perform their jobs better.” Perceived ease of use refers to “whether an application is easy to use, meaning the performance benefits are not outweighed by the effort of using the application” (Davis, 1989). The TAM suggests that perception of usefulness and ease of use about new technology are beliefs that inspire a consumer’s attitude toward using that technology (Davis, 1989). The application of TAM has become increasingly popular in various domains to understand consumer technological innovation adoption (Venkatesh, 2000). Previous studies have indicated that the TAM plays a crucial role in defining users’ acceptance of VR (Huang et al., 2016; Manis and Choi, 2019). TAM has also extended by adding external variables (Venkatesh, 2000), especially technological variables. Kim et al. (2020a) revealed that the authenticity experience in VR presentations plays a positive role in consumers’ behavioral intention.

Research on information technology has also explained that emotions, such (Huang et al., 2013), enjoyment (Huang et al., 2013), emotional involvement (EI) (Hirschman and Holbrook, 1982; Laurent and Kapferer, 1985), and flow (Csikzentimihalyi, 1975), are the key hedonic antecedents to understand human-technology interaction. In line with this, Kim et al. (2020a) conceptualized enjoyment, EI, and flow state as affective responses to predict travel intention through VR demonstrations. Previous studies have shown that VR authenticity and efficiency induce affective responses, directly influencing consumer behavior (Huang et al., 2016; Yung and Khoo-Lattimore, 2019; Kim et al., 2020a). Numerous studies have shed light on consumer response to VR technology and its importance in the context of marketing (Loureiro et al., 2019; Martínez-Navarro et al., 2019; Wedel et al., 2020). In addition, other scholars called for additional empirical research on customer VR experience in developing countries to identify the key factors that influence consumer behavior. Most of the related previous studies have focused on adoption intention or willingness to purchase VR services (Tavakoli and Mura, 2015; Loureiro et al., 2019; Kim et al., 2020a), although most VR studies on behavioral intentions are one dimensional. However, evidence on the potential factors that trigger those drivers that young generation to be loyal customers of VR services despite the relatively high price premium has not been answered well in the existing literature. Therefore, the current research is a pilot empirical study that aims to fill this gap.

The current research may have three contributions to the body of knowledge on VR services. First, we combine the TAM dimensions and authenticity experience of VR to support that TAM dimensions and authenticity experience positively contribute to customer loyalty in VR-enabled services via affective responses. When users experience authentic VR presentation, beneficial and easy, consumer emotional responses are provoked, thus influencing customer behavioral intentions. Second, we introduce affective responses to unveil the mechanism of customer loyalty in VR-enabled services. We verified that affective responses, such as enjoyment, EI, and flow state, significantly mediate the relationships between the drivers (i.e., TAM dimensions and authenticity) and customer loyalty [i.e., continued use, recommendation, and willingness to pay premium (WPP)]. Third, scarce research focused on customer loyalty behaviors in the VR domain; to the best of our knowledge, this study is the first to investigate customer loyalty to VR-enabled services, going beyond most research on adoption purchase intention (Tavakoli and Mura, 2015; Loureiro et al., 2019; Kim et al., 2020a), and VR technology adoption in the context of marketing (Loureiro et al., 2019; Martínez-Navarro et al., 2019; Wedel et al., 2020). The study also provides insights for VR practitioners and businesses. Managers who want to introduce VR services in their service landscapes should design unique VR content which is appealing and enhances consumer perception of authenticity in VR-enabled services.

## Literature Review

### Authenticity of Virtual Reality

Gilmore and Pine (2007) explained authenticity as “the extent of consumer sensibility perceptions to which experiences, services, or products are novel, authentic, original, exceptional, or unique.” The authenticity experience obtained from technology use boosts the behavioral intention of users (Kim et al., 2020b). For instance, VR services made VR heritage sites convenient for customers to enjoy attractiveness by enhancing authenticity and visitor intention to the places (Dueholm and Smed, 2014). Similarly, in VR learning, a high level of authenticity results in an excellent level of immersion (Lan and Liao, 2018). For example, Loup et al. (2016) revealed that learners obtain high immersion in VR due to the authenticity experience, enhancing user engagement. By contrast, Guttentag (2010) demonstrated that VR travel activities might not be realistic because of the low level of technology use, influencing user authentic VR experience. However, the critical role of authenticity experience in research related to VR, the investigation is not sufficient to address this concept in the context of VR-enabled services (Kim et al., 2020a). Therefore, to understand the effect of authenticity on consumer behavior, the current study investigates the role of authenticity in VR-enabled services based on TAM.

### A General Comparison With Previous Studies

First, most prior research focused on consumers’ adoption of VR-enabled services in various industries adoption (Huang et al., 2013; Tussyadiah et al., 2018; Li and Chen, 2019; Kim et al., 2020a). A recent study on VR devices by Lee et al. (2019) suggested that VR ease of use and usefulness influence customer motivation toward technology adoption. Similarly, Huang et al. (2016) examined tourist intention to travel destinations in VR and suggested that technology variables influence individuals’ adoption behavior. VR specialized services are effective in marketing (Loureiro et al., 2019) because specialized services can boost consumer experience (Jung et al., 2016). For instance, Tussyadiah et al. (2018) investigated the application of VR marketing and found positive effects on consumer attitudes and behavioral intention. However, scarce of them paid attention to loyalty behavior with VR-enabled services. As to our knowledge, there is no study on continued use, recommendation and WPP in the context of VR-enabled services. Second, most au applied the TAM model to explain consumer adoption, neglecting the unique utility of VR-authenticity experience (Lee et al., 2019; Luna-Nevarez and McGovern, 2021). Third, we introduced and verified the complete three dimensions of affective response are mediators, while the prior research examined only attachment (Kim et al., 2020a). Table 1 summarizes the literature.

**TABLE 1:** Previous research on the predictors and outcomes of VR adoption intention/VR purchase intention.

Authors	Predictors	Outcomes
Kim et al. (2020b)	Subjective wellbeing, authentic experience	Behavioral intention
Marasco et al. (2018)	Visual appeal, emotional involvement	Behavioral intention to visit/revisit the cultural heritage site
Huang et al. (2016)	Ease of use, usefulness, autonomy, competence, relatedness	Enjoyment, behavioral intention
Li and Chen (2019)	Ease of use, usefulness, enjoyment	Travel intention
Kim and Ko (2019)	Telepresence, flow experience	Satisfaction
Martínez-Navarro et al. (2019)	Presence, brand recall	Purchase intention
Manis and Choi (2019)	Ease of use, usefulness, enjoyment, attitude	Purchase intention, use intention
Beck and Crié (2018)	Virtual Fitting Room on the website	Purchase intention
Luna-Nevarez and McGovern (2021)	Ease of use, usefulness, enjoyment, immersion, trust, VR self-efficacy	Intentions
Kim et al. (2020a)	Cognitive and affective response	attachment and visit intention
This study	Authenticity, affective responses (i.e., enjoyment, Emotional involvement, and flow state)	Customer loyalty (i.e., continued use, recommendation and willingness to pay premium)

### Affective Responses in Virtual Reality-Enabled Services

Research on emotions, affections or hedonic consumption in marketing literature started early in 1970 (Hirschman and Holbrook, 1982; Huang et al., 2013). Peterson et al. (1986) described affections as experienced emotions and feelings of individuals. Holbrook and Hirschman (1982) defined emotions (affective responses) as aspects of individuals’ or customers’ behavior concerned with fantasy and emotive perceptions concerning the usage of products and experiences. Kim et al. (2020a) asserted that constructs, such as EI, enjoyment, and flow state, positively influence users’ visit intention and attachment toward destinations experienced via VR; they also conceptualized these emotions as sub-constructs of affective responses.

A review of previous VR studies also revealed that enjoyment (Roca and Gagné, 2008), EI (Holsapple and Wu, 2007), and flow experience (Kim and Hall, 2019) are significant predictors of consumers’ participation in virtual environments.

**FIGURE 1:**
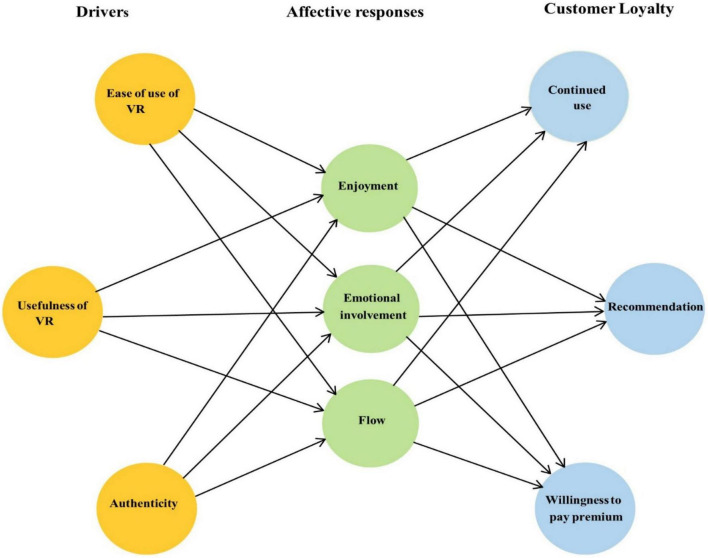
Framework of the current research.

Therefore, based on previous evidence, we operationalized constructs, such as EI, enjoyment, and flow state, in our study of affective responses (Kim et al., 2020a) (see Figure 1). Enjoyment, a subcontract of affective responses, is considered an enjoyable, pleasurable variable described by Venkatesh (2000) as an activity related to particular system use and is believed to be pleasure arousing in its own way apart from the performance consequences generated from the use of the system. Van der Heijden (2004) indicated that the degree to which users experience fun while interacting with hedonic technology could be specified by enjoyment. Huang et al. (2013) investigated that enjoyment perception greatly influences users’ behavior in 3D virtual site experience and mobile social media sites (Kim et al., 2017).

Emotional involvement is defined as “the extent to which customer emotionally involved in a behavior” (Holsapple and Wu, 2007). A study examined hedonic behavior in a 3D virtual environment and proposed that EI is key in exploring user experience (Saeed et al., 2009). For instance, a higher comprehension of EI in a VR tourism site is interrelated with the high behavioral intention to visit the tourist spot (Huang et al., 2013). In addition, as a positive affective response, EI encourages the perception that VR experience is genuine, resulting in intention to visit the target destination portrayed in VR (Guttentag, 2010).

Flow state is explained as “the holistic excitement that individual feel when acting with total engagement” (Csikszentmihalyi, 2000) and reported as the optimal experience. Flow state in a 3D virtual setting greatly determines learners’ attitudes toward e-learning (Huang et al., 2010). Flow state is an essential tool to identify user experiences in VR services, impact involvement, and likely VR tourists’ behavior (Huang et al., 2012). In VR services, flow state has a substantial influence on easiness and behavioral intention to travel (Huang et al., 2013) and word of mouth (WOM) (Gao et al., 2014). In advanced technologies, flow state is explained as captivation, immersion, profound involvement, and an emphasis on the application of technologies (Kim et al., 2017). Although previous research has verified the effectiveness of flow state, EI, and enjoyment in understanding consumer behavior and interaction with new technologies (Huang et al., 2013; Li and Chen, 2019; Kim et al., 2020a), studies that examined the influence of affective responses on behavioral intention.

## Hypothesis Development

### Technology Acceptance Model Variables and Affective Responses in Virtual Reality-Enabled Services

#### Enjoyment

The concept of enjoyment is broadly discussed in consumer behavior and considered the critical factor in understanding users’ virtual experience. Venkatesh (2000) described enjoyment as an activity related to particular system usage, and it is believed to be pleasure arousing apart from the performance consequences. Numerous studies have revealed that the usefulness and ease of use of VR have a positive association with enjoyment in terms of virtual experience (Huang et al., 2013; Huang et al., 2016; Kim and Hall, 2019). Recent evidence suggested that the usefulness and easiness of VR contribute to user enjoyment enhancement (Li and Chen, 2019). Yang et al. (2016) explained that potential users receive pleasure from experiencing virtual products, such as wearable devices. According to researchers, consumers received helpful information about real destinations using VR services, making trip planning decisions easy. Moreover, researchers verified that user enjoyment is provoked by the degree of VR easiness and usefulness (Li and Chen, 2019).

#### Emotional Involvement

Marketing literature has attracted considerable attention to customer EI in VR technology (Marasco et al., 2018). EI is the degree to which a user is engaged emotionally in an activity. Saeed et al. (2009) asserted that EI is an important factor in understanding user experience in the entertainment nature of technology. A recent study indicated that immersive 3D experience affects users’ emotional state when experiencing VR devices (Marasco et al., 2018). Immersive VR content presentation stimulates the users’ feeling of being there and boosts their EI (Gutierrez et al., 2008; Guttentag, 2010). Scholars have examined the effect of user attributes in experiencing new technology and found a positive association between EI and perceived usefulness and easiness (Doherty et al., 2006). Huang et al. (2013) investigated the effect of VR tourism on consumer intention. They claimed that the ease of use and usefulness of VR induce user emotion to visit the destinations presented in VR.

#### Flow State

Flow state has been used as a theoretical framework to understand user interaction with technology; previous studies have revealed that flow state is a vigorous construct in VR consumption (Wu et al., 2013). Research exploring user behavior in virtual tourism suggests that customers participate in VR demonstration as an innovative technology for hedonic stimuli, such as flow state (Kim et al., 2020a). A recent study on an online game showed that players’ easiness positively affects joy, which further stimulates the consumers’ flow state (Lowry et al., 2013). A recent study indicated that perceived usefulness significantly influences flow state, affecting customers’ online impulse purchasing in impulse shopper and system users (Wu et al., 2016). Another study applied TAM and flow experience in VR services; results revealed that perceived usefulness is associated with flow state (Huang et al., 2013). Finally, Kim and Hall (2019) indicated that perceived ease of use and usefulness are associated with flow state in exploring the consumer’s intention to use 3D virtual world. In line with the literature, the following hypotheses are proposed:

H1: Ease of use of VR has a positive effect on consumers’ (a) enjoyment, (b) emotional involvement, and (c) flow state while experiencing VR-enabled services.

H2: Usefulness of VR has a positive effect on consumers’ (a) enjoyment, (b) emotional involvement, and (c) flow state while experiencing VR-enabled services.

### Authenticity Experience and Affective Responses in Virtual Reality-Enabled Services

Authenticity plays a vital role in enhancing customer VR experiences. Authenticity is explained as “the extent of consumer sensibility perceptions to which experiences, services, or products are novel, real, original, exceptional, or unique” (Gilmore and Pine, 2007). Dueholm and Smed (2014) revealed that authentic experience provides fun to visitors to engage and move away from their daily activities, thereby leading to enjoyment. In addition, research on hedonic experiences of VR verified that a sense of authenticity in VR content boosts users’ enjoyment, attitudes, and visiting intentions (Tussyadiah et al., 2018).

Similarly, VR tourism induces users’ emotional responses and perception of authentic experience, which influences the customer intention to the destination, signifying that authentic experience has a positive and significant effect on consumer EI and behavior (Guttentag, 2010; Kim et al., 2020a). Similarly, Baños et al. (2004) examined immersion and emotions in VR and claimed that the customers’ feeling of being there influences customers’ EI. Subsequently, perceived authenticity is triggered by VR experience involving all senses, especially the visionary element has a significant role in the perception of authenticity that impacts the flow state of users (Mura et al., 2017). Thus, the following hypotheses are proposed.

H3: Authenticity experience of VR-enabled services has a positive effect on consumers: (a) enjoyment, (b) emotional involvement, and (c) flow.

### Affective Responses and Customer Loyalty

Oliver (1999) explained loyalty as “a profoundly held devotion to rebuy or patronize a preferred product/service constantly in the future (p. 34).” Jugenheimer (1979) argued that customer loyalty consists of several behavioral components. Based on the studies of Zeithaml et al. (1996) and Oliver (1999), the dimensions of behavioral intentions, we conceptualized customer loyalty as three sub-dimensions, including continued use, recommendation intention, and willingness to pay premium.

Continued use is defined as the consumers’ reuse of information systems after their first experience, which is essential in continued use (Bhattacherjee, 2001). A recommendation is defined as the psychological behavior that encourages family members, colleagues, and friends to use their experienced products and services (Prayag et al., 2017). Willingness to pay (WTP) is described as the total sum of money a shopper is keen to spend for a specific service or product (Cameron and James, 1987). Price premiums, referred to as “excessive amount paid over the average price that is adequate by the actual value of the product and services” (Rao and Bergen, 1992), suggest an indicator of consumer willingness to pay (Vlosky et al., 1999). Thus, WPP is the extra amount a consumer is willing to spend over a fair price of a product and service.

Previous empirical studies have indicated that enjoyment is a decisive factor of users’ continued use of VR games (Jang and Park, 2019). Similarly, a study that explored users’ motivation to continue playing AR Pokémon Go revealed that enjoyment positively influences the players’ intention for continued use (Ghazali et al., 2019). In addition, Huang et al. (2017) revealed that an enjoyable online game experience motivates users to recommend products or services to their colleagues.

Another study on VR tourism using VR wearable devices indicated that users’ EI influences to visit/revisit intention to destination (Marasco et al., 2018). A study on the emotions and intention to recommend among virtual atmospheric cues revealed that emotional factors significantly affect recommendation in both genders (Loureiro and Ribeiro, 2014). Flavián et al. (2019b) found that VR services induce more immersive experiences, advanced sensory stimulation, engagement, and more excellent behavioral intentions to the destination.

Kim and Hall (2019) revealed that visitors are more likely to continue visiting a tourism destination due to the flow state in VR tourism. Furthermore, research has shown that the flow state of visitors positively influences recommendation intention in the context of a music festival (Ding and Hung, 2021).

A study that examined the user experience of immersion toward AR games similar to VR revealed that hedonism, which indicates enjoyment and pleasure of using a specific technology, increases users’ satisfaction when using AR (Shin, 2019), satisfaction is closely related to affective response (Kim et al., 2020a), applied to our study consumer might be willing to pay premium for VR-enabled services based on their emotional experience. Thus, on the basis of the literature, the following hypotheses are suggested:

H4: Enjoyment positively influences: (a) continued use and (b) recommendation of VR-enabled services.

H5: Emotional involvement positively influences: (a) continued use, (b) recommendation, and (c) willingness to pay premium of VR-enabled services

H6: Flow positively influence: (a) continued use and (b) recommendation of VR-enabled services.

### Mediation Effect of Affective Responses (Enjoyment, Emotional Involvement, and Flow)

Several studies have considered enjoyment an essential intrinsic and hedonic motivation construct in adopting IT systems and services (Venkatesh, 2000; Van der Heijden, 2004). Customer-perceived enjoyment plays mediating role in the relationship between VR technology and behavioral intention. Jang and Park (2019) indicated that enjoyment plays a mediating role between the functional aspect of VR games and continued use. A recent study on the determinant of the continuous playing of Pokémon Go suggested that enjoyment plays a mediating role (Ghazali et al., 2019). In VR tourism marketing, Huang et al. (2013) suggest that consumer emotional involvement influences users’ intention to destination in the future and further revealed that consumer EI plays mediating role between TAM and intention to destination in the future. Kim et al. (2017) studied the effect of users’ intrinsic and extrinsic motivation of purchase intention in the traveling behavior of senior users of social network sites; they suggested that flow state show a significant mediation affect between perceived usefulness and intention.

Flow state plays a mediating role between TAM and continued use in the context of VR tourism (Kim and Hall, 2019). Similarly, previous research has predicted that the flow state plays a mediation role between the function component of technology and consumer behavior (Chen et al., 2017). EI and flow state were studied in a 3D virtual world in travel and tourism. Results confirmed that EI and flow state play a mediating role in the relationship between perceived ease of use and perceived usefulness and consumer intention (Huang et al., 2013). Kim et al. (2020a) used the S-O-R model to explore consumer behavior in VR tourism. Their examination of the authentic experience of VR in tourism indicated that affective responses, such as enjoyment, EI, and flow state, play a mediating role in predicting consumers’ behavioral intention. Based on the above literature, we propose the following hypotheses:

*H7*-a, *H8*-a, and *H9*-a: While experiencing VR-enabled services, enjoyment, emotional involvement, and flow mediates the effect of ease of use on (a) continued use, (b) recommendation, and (c) willingness to pay premium.

*H7*-b, *H8*-b, and *H9*-b: While experiencing VR-enabled services, enjoyment, emotional involvement, and flow mediates the effect of usefulness on (a) continued use, (b) recommendation, and (c) willingness to pay premium.

*H7*-c, *H8*-c, and *H9*-c: While experiencing VR-enabled services, enjoyment, emotional involvement, and flow mediates the effect of authenticity on (a) continued use, (b) recommendation, and (c) willingness to pay premium.

## Methodology

The authors adopted a self-administered survey approach and random sampling technique. Data were collected between December 2020 and February 2021. The participants were approached in VR studios at several big shopping malls in four big cities of Pakistan. Data were collected from volunteers of VR users in VR studios, and participants were requested to fill the survey based on their VR experience using VR devices/gadgets in the studio. We only included those respondents who gave confirmative answers to our filter questions. VR studios provide the most immersive and interactive VR experience, from taking on adventurous missions to exploring the incredible world and experiencing the impossible; users could experience various VR-immersive services, including VR games, VR music shows, VR sports, VR movies, and VR real estate. For instance, Retina VR, which introduces VR real estate tours, allow individuals to search properties anytime without leaving their home.

Respondents were asked a filter question, i.e., ‘‘Have you experienced any of the VR services^1^ ? The affirmative respondents were included in the study. The response rate was 71% (320 valid questionnaires out of 450). Researchers followed the rule of thumb based on Hair et al. (2010)’s suggestion, that is, 10 times the observation of the number of item of variables to be analysed; thus, the required sample size for this research should be at least 320 (32 items, 10 cases). Churchill (1991) recommended that the number of surveys in consumer studies should range between 200 and 500 responses. Therefore, we assumed that 320 valid questionnaires would be sufficient to analyse the data for this research. A statistical analysis of valid samples was conducted by using SPSS 24, and the results are reported below. The sample consisted of 57% males and 43% females. A large portion (85%) of the sample belongs to the 18–25 years age group; approximately 9% of the participants were between 26 and 30 years old, 2% of the respondents were above 31 years old, and 4% of respondents was below 18 years old. A total of 72% completed their bachelor’s degree, 19% had completed their master’s degree, and 5% had completed their doctoral degree. The monthly income profile of the sample showed that 76 (24%) respondents had an income ranging from PKR 1 to 15,000 ($85); 128 (40%) had an income ranging from PKR 16,000 to 25,000 ($91 to $142); 80 (25%) had an income ranging from PKR 26,000 to 35,000 ($148 to $200); 36 (11%) had an income ranging from PKR 36,000 ($205) and above.

The researchers used survey questionnaires to investigate the hypotheses introduced in this study. The definitions of all nine variables are shown in Table 2. The last section presents the participants’ demographic information, such as age, gender, education, and monthly income. A seven-point Likert scale ranging from 1(strongly disagree) to 7(stronger agree) was used to measure all items.

**TABLE 2:** Definitions of study variables.

Constructs	Items			Sources
Ease of use of VR	EU1	Learn to use VR-enabled services was	Difficult/easy	Davis, 1989
	EU2	My interaction with VR-enabled services was	Not flexible/flexible	
	EU3	I find VR-enabled services	Difficult to use/easy to use	
	EU4	To become skillful, I find VR-enabled services	Difficult/easy	
Usefulness of VR	USF1	VR-enabled services are	Useless/useful	Davis, 1989
	USF2	Using VR-enabled services may achieve things that important to me	Decrease chances/increase chances	
	USF3	Using VR-enabled services helps me accomplish things	Slowly/quickly	
	USF4	Using VR-enables services may	Decreases my productivity/increases productivity	
Authenticity	AUT1	Using VR-enables services provided me experience	Unauthentic/Authentic	Kim et al., 2020a
	AUT2	Using VR-enables services provided me experience	Unreal/genuine	
	AUT3	Using VR-enables services provided me experience	Normal/exceptional	
	AUT4	Using VR-enables services provided me experience	Usual/Unique	
Enjoyment	ENJ1	I enjoyed experiencing the VR-enabled services very much	Huang et al., 2013	
	ENJ2	I think the feeling in 3D as virtual enabled services was pretty enjoyable		
	ENJ3	I would describe the experience very interesting		
	ENJ4	The experience was fun		
Emotional involvement	EI1	While I was experiencing VR-enabled devices, I feel carried off by the VR devices	Saeed et al., 2009	
	EI2	While I was experiencing VR-enabled device I feel as if I was part of the VR environment		
	EI3	When I was experiencing I feel deeply involved in the VR environment		
Flow	F1	When experiencing wearable VR-enabled services, my attention was totally focused	Trevino and Webster, 1992	
	F2	Experiencing VR-enabled services excites my curiosity		
	F3	Experiencing VR-enabled services was intrinsically interesting		
	F4	When experiencing VR-enabled services, I feel in control		
Continued use	CU1	I will continue to use VR-enabled services in the future	Kim and Hall, 2019	
	CU2	I will update VR-enabled related services in the future		
	CU3	I will search for VR-enabled services in the future		
Recommendation	RC1	I would like to recommend VR-enabled devices to others	Brown et al., 2005	
	RC2	I would like to share this experience with others		
	RC3	I would like to encourage others to use VR-enabled services		
Willingness to pay premium	WPP1	I would pay more for VR-enabled services	Netemeyer et al., 2004; Laroche et al., 2001	
	WPP2	I am willing to spend more money in order to experience VR-enabled services		
	WPP3	It is acceptable to pay extra for experiencing VR-enabled services		

## Results

### Measurement Model

Data were analysed using a two-step approach (Anderson and Gerbing, 1988) via smart PLS. The measurement of composite reliability and Cronbach’s alphas are shown in Table 3. The average variance extracted (AVE) and factor loading were checked for discriminant and convergent validity. The alpha value was above 0.70 of all the nine constructs. The composite reliability of maximum constructs was greater than 0.60 (Bagozzi and Youjae, 1988). The instrument was reliable for the measurement of the latent construct. The standardized factor loadings of almost all items were above the standard value of 0.70 and were significant at the 0.01 level of significance, although two items with a value of 0.67 and 0.68 were acceptable and satisfied convergent validity. All AVE values were above the minimum threshold of 0.50, implying that maximum variance is interpreted with constructs (Fornell and Larcker, 1981). A study instrument indicated a high degree of validity and reliability for the function of the latent construct.

**TABLE 3:** Reliability testing and convergent validity.

Construct	Item	Loading	CR	AVE	Cronbach’s alpha
Ease of use	EU1	0.823	0.894	0.679	0.842
	EU2	0.806			
	EU3	0.851			
	EU4	0.816			
Usefulness	USF1	0.792	0.897	0.685	0.846
	USF2	0.849			
	USF3	0.865			
	USF4	0.802			
Authenticity	AUT1	0.850	0.915	0.730	0.877
	AUT2	0.828			
	AUT3	0.890			
	AUT4	0.849			
Enjoyment	ENJ1	0.885	0.928	0.763	0.896
	ENJ2	0.872			
	ENJ3	0.878			
	ENJ4	0.859			
Emotional involvement	EI1	0.895	0.928	0.763	0.896
	EI2	0.916			
	EI3	0.918			
Flow	F1	0.842	0.972	0.632	0.803
	F2	0.830			
	F3	0.831			
	F4	0.664			
Continued use	CU1	0.857	0.801	0.765	0.852
	CU2	0.901			
	CU3	0.880			
Recommendation	R1	0.839	0.802	0.766	0.847
	R2	0.894			
	R3	0.892			
Willingness to pay premium	WPP1	0.926	0.837	0.841	0.906
	WPP2	0.929			
	WPP3	0.896			

*CR, composite reliability; AVE, Average variance extracted.*

Moreover, the AVE value and latent variable correlation coefficient matrix were used to evaluate discriminant validity. All the AVE were higher than 0.5 and even higher than the square root of the matrix shown in Table 4. Therefore, the discriminant validity of the current research is acceptable (see Table 4).

**TABLE 4:** Correlation of discriminant validity.

	EU	USF	AUT	ENJ	EI	Fl	CU	REC	WPP
EU	**0.82**								
USF	0.392***	**0.83**							
AUT	0.527***	0.489***	**0.85**						
ENJ	0.368***	0.405***	0.579***	**0.87**					
EI	0.349***	0.404***	0.613***	0.677***	**0.87**				
Fl	0.364***	0.436***	0.530***	0.798***	0.666***	**0.79**			
CU	0.097***	0.036***	0.224***	0.381***	0.417***	0.066***	**0.87**		
REC	0.350***	0.347***	0.392***	0.646***	0.509***	0.639***	0.119***	**0.88**	
WPP	0.232***	0.338***	0.288***	0.381***	0.344***	0.407***	0.057***	0.053**	**0.97**
Mean	5.25	5.28	5.45	5.54	5.06	5.53	5.22	5.18	4.48
Standard deviation	1.23	1.024	1.17	1.084	1.18	1.26	1.34	1.22	1.23

*Table values denote correlation is significant at the 0.01 level of significant; the square roots of AVE values are shown on the main diagonal for the reflective scales, while the rest are correlation coefficient of latent variables.*

### Predictive Relevance

**FIGURE 2:**
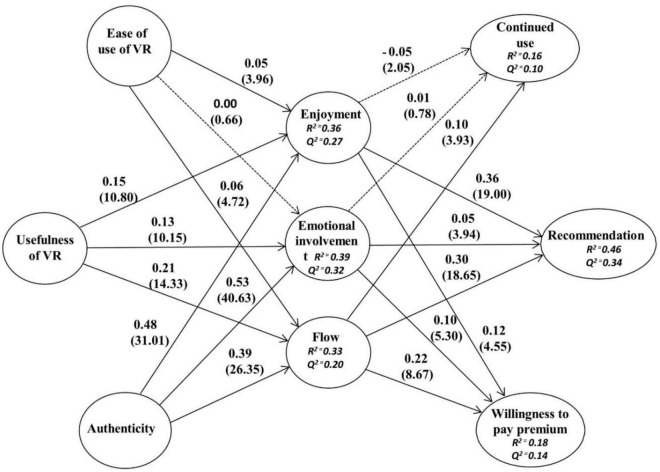
Results of path analysis main effects. The dotted arrow shows the non-significant path coefficient, and *t*-values are presented in parentheses.

The coefficient of determination (*R*^2^) and cross-validated redundancy (*Q*^2^) was examined for inner model assessment. *R*^2^ is classified into three types: high, moderate, and low (Sanchez, 2013). If *R*^2^ is more than 0.6, then it is high; if *R*^2^ is within 0.3–0.6, then it is moderate; if it is below 0.3, then it is low. *R*^2^ values in Figure 2 show the model fit. The blindfolding method was applied to check *Q*^2^. It evaluates the predictive relevance of the inner model (Joe et al., 2014). *Q*^2^ value should be greater than zero. All the *Q*^2^ values in Figure 2 are greater than zero, thus, confirming the fitness of the model.

### Common Method Bias

Data were collected from the same sources for all measurements, leading to a common bias due to the method or the measurement instrument. Harman’s signal factor test was conducted to avoid common method bias on all items of this research (Podsakoff et al., 2003) were tested by using SPSS 24. The results indicated that six factors whose eigenvalues were greater than 1, as explained by exploratory factor analysis, accounted for 67.88% of variances. The first single factor extracted only 34.45% of the total variance, which is far less than 50%; therefore, the study has no possible threat of common method bias.

### Hypotheses Testing

The structural equation model was used to investigate the proposed hypotheses. The results of the tested hypotheses are shown in Table 5 and Figure 2. Results show that the drivers (ease of use, usefulness, and authenticity) of VR significantly affect affective responses. According to the results, ease of use and usefulness have a significant effect on enjoyment. Thus, H1a EU → ENJ (β = 0.05, *t* = 3.96) and H2a USF → ENJ (β = 0.15, *t* = 10.80) were supported. Our results are similar to previous studies conducted by Venkatesh (2000) and Van der Heijden (2004). Similarly, usefulness shows a significant effect on EI, thus, confirming H2b USF → EI (β = 0.13, *t* = 10.15). This finding is consistent with Saeed et al. (2009) and Huang et al. (2013). Moreover, ease of use and usefulness have a significant effect on the flow state. Thus, H1c EU → FL (β = 0.06, *t* = 4.72) and H2c USF → FL (β = 0.21, *t* = 14.33) are supported. These results are consistent with the result of Kim and Hall (2019) in the tourism context and Wu et al. (2016) in online impulse buying. In addition, authenticity has a significant effect on affective responses, confirming H3a AUTH → ENJ (β = 0.48, *t* = 31.01), H3b AUTH → EI (β = 0.53, *t* = 40.63), and H3c AUTH → FL (β = 0.39, *t* = 26.35). The results of the current study are similar to those of Guttentag (2010); Kim et al. (2020a), and Tussyadiah et al. (2018) in the context of tourism.

**TABLE 5:** Hypotheses testing.

Hypothesized path	PC	*t*-V	Support
H1a: EU → ENJ	0.05	3.96	Yes
H1b: EU → EI	0.00	0.66	No
H1c: EU → FL	0.06	4.72	Yes
H2a: USF → ENJ	0.15	10.80	Yes
H2b: USF → EI	0.13	10.15	Yes
H2c: USF → FL	0.21	14.33	Yes
H3a: AUT → ENJ	0.48	31.01	Yes
H3b: AUT → EI	0.53	40.63	Yes
H3c: AUT → FL	0.39	26.35	Yes
H4a: ENJ → CU	−0.05	2.05	No
H4b: ENJ → REC	0.36	19.00	Yes
H4c: ENJ → WPP	0.12	4.55	Yes
H5a: EI → CU	0.01	0.78	No
H5b: EI → REC	0.05	3.94	Yes
H5c: EI → WPP	0.10	5.30	Yes
H6a: FL → CU	0.10	3.93	Yes
H6b: FL → REC	0.30	18.65	Yes
H6c: FL → WPP	0.22	08.67	Yes

*EU, Ease of use; USF, Usefulness; AUT, Authenticity; ENJ, Enjoyment; EI, Emotional involvement; FL, Flow; CU, Continued use; REC, Recommendation; WPP, willingness to pay premium PC, path coefficient; t-V, t-Value.*

Affective responses have a significant influence on continued use, recommendation, and WPP.

Therefore, H4b ENJ → REC (β = 0.36, *t* = 0.19), H5b EI → REC (β = 0.05, *t* = 3.94), and H6b FL → REC (β = 0.30, *t* = 18.65) are supported. Affective responses have a positive effect on WPP. Thus, H4c ENJ → WPP (β = 0.12, *t* = 4.55), H5c EI → WPP (β = 0.10, *t* = 5.30), and H6c FL → WPP (β = 0.22, *t* = 08.67) are also supported. Flow state affects continued use, thus, confirming H6a FL → CU (β = 0.10, *t* = 3.93). Our result is consistent with that of Kim and Hall (2019). By contrast, ease of use had no direct effect on EI; thus, H1b EU → EI (β = 0.00, *t* = 0.66) is rejected. This result contrasts with the study of Doherty et al. (2006). In addition, enjoyment has no direct influence on continued use; therefore, H4a ENJ → CU (β = −0.05, *t* = 2.05) are rejected. This hypothesis contrasts with a previous study by Ghazali et al. (2019). EI also has a non-significant effect on continued use; thus, H5a EI → CU (β = 0.01, *t* = 0.78) is rejected. This result is consistent with a previous study conducted by Marasco et al. (2018).

### Mediation Effects

We employed the bootstrapping method to check the mediation effect of affective responses. Results (in Table 6) show that enjoyment mediates the relationship between ease of use and recommendation (*z* = 3.76, *p* = 0.000) and WPP (*z* = 2.89, *p* = 0.001). Similarly, enjoyment has a significant mediation effect between usefulness and recommendation (*z* = 9.38, *p* = 0.000) and WPP (*z* = 4.21, *p* = 0.000). In addition, enjoyment has a significant mediating role (*z* = 1.96, *p* = 0.049), between authenticity and continued use, recommendation (*z* = 16.47, *p* = 0.000), and WPP (*z* = 4.53, *p* = 0.000). Thus, enjoyment show a significant mediation effect between authenticity and continued use, recommendation, and WPP. By contrast, enjoyment has an non-significant mediation effect between drivers of VR ease of use (*z* = 1.77, *p* = 0.07), usefulness (*z* = −1.93, *p* = 0.053), and continued use.

**TABLE 6:** Mediation effect.

Predictor	Mediator	Outcome	*Z* Value	*P* Value	Status
EU	ENJ	CU	1.773	0.076	Rejected
EU	ENJ	REC	3.988***	0.000	Accepted
EU	ENJ	WPP	3.045*	0.002	Accepted
EU	EI	CU	0.509	0.611	Rejected
EU	EI	REC	0.662	0.508	Rejected
EU	EI	WPP	0.668	0.504	Rejected
EU	FL	CU	0.978	0.328	Rejected
EU	FL	REC	4.519***	0.000	Accepted
EU	FL	WPP	4.129***	0.000	Accepted
USF	ENJ	CU	–1.936	0.053	Rejected
USF	ENJ	REC	9.382***	0.000	Accepted
USF	ENJ	WPP	4.214***	0.000	Accepted
USF	EI	CU	0.776	0.438	Rejected
USF	EI	REC	3.420*	0.001	Accepted
USF	EI	WPP	4.627***	0.000	Accepted
USF	FL	CU	0.998	0.318	Rejected
USF	FL	REC	11.490***	0.000	Accepted
USF	FL	WPP	7.612***	0.000	Accepted
AUT	ENJ	CU	1.965***	0.049	Accepted
AUT	ENJ	REC	16.470***	0.000	Accepted
AUT	ENJ	WPP	4.535***	0.000	Accepted
AUT	EI	CU	0.778	0.437	Rejected
AUT	EI	REC	3.60***	0.000	Accepted
AUT	EI	WPP	5.106***	0.000	Accepted
AUT	FL	CU	0.999	0.318	Rejected
AUT	FL	REC	14.989***	0.000	Accepted
AUT	FL	WPP	8.411***	0.000	Accepted

*p < 0.1; **p < 0.05; ***p < 0.01. EU, Ease of use; USF, Usefulness; AUT, Authenticity; ENJ, Enjoyment; EI, Emotional involvement; FL, Flow; CU, Continued use; REC, Recommendation; WPP, willingness to pay premium.

Note: EU = EI has a significant mediation effect between usefulness and recommendation (*z* = 3.42, p = 0.001) and WPP (*z* = 4.62, p = 0.000). Similarly, EI had significant mediation effect between authenticity and recommendation (*z* = 3.60, *p* = 0.001) and WPP (*z* = 5.10, *p* = 0.000). By contrast, EI insignificantly mediates the relationship between ease of use and customer loyalty dimensions including continued use (*z* = 0.50, *p* = 0.61), recommendation (*z* = 0.62, *p* = 0.50), and WPP (*z* = 0.66, *p* = 0.50). In addition, EI shows a non-significant relationship between VR driver usefulness (*z* = 0.77, *p* = 0.43), authenticity (*z* = 0.77, *p* = 0.43), and continued use. Flow state has a mediation effect between ease of use and recommendation (*z* = 4.51, *p* = 0.000) and WPP (*z* = 4.12, *p* = 0.001). Similarly, flow state has a mediation effect between usefulness and recommendation (*z* = 11.49, *p* = 0.000) and WPP (*z* = 7.61, *p* = 0.001). In addition, flow state has a significant mediation effect between authenticity of VR and recommendation (*z* = 14.98, *p* = 0.000) and WPP (*z* = 8.41, *p* = 0.000). By contrast, flow state shows non-significant effect between VR drivers = ease of use (*z* = 0.97, *p* = 0.32), usefulness (*z* = 0.99, *p* = 0.31), authenticity (*z* = 0.99, *p* = 0.31), and continued use.

Flow state has a mediation effect between ease of use and recommendation (*z* = 4.51, *p* = 0.000) and WPP (*z* = 4.12, *p* = 0.001). Similarly, flow state has a mediation effect between usefulness and recommendation (*z* = 11.49, *p* = 0.000) and WPP (*z* = 7.61, *p* = 0.001). In addition, flow state has a significant mediation effect between authenticity of VR and recommendation (*z* = 14.98, *p* = 0.000) and WPP (*z* = 8.41, *p* = 0.000). By contrast, flow state shows non-significant effect between VR drivers = ease of use (*z* = 0.97, *p* = 0.32), usefulness (*z* = 0.99, *p* = 0.31), authenticity (*z* = 0.99, *p* = 0.31), and continued use.

## Control Variable Test

The four demographic variables were controlled using smart Smart PLS 3 to check the relationships with continued use, recommendation, and WPP. According to the results, age has a non-significant effect on continues usage (β = 0.10, *p* = 0.16), recommendation (β = 0.09, *p* = 0.31), and WPP (β = 0.12, *p* = 0.18). Similarly, gender has a non-significant effect on continues usage (β = 0.09, *p* = 0.16), WPP (β = 0.01, *p* = 0.74), and significant effect on recommendation (β = 0.13, *p* = 0.034). Similarly, income has a non-significant effect on continues usage (β = 0.05, *p* = 0.43), recommendation (β = 0.00, *p* = 0.91), and WPP (β = −0.02, *p* = 0.70). In addition, education has a non-significant effect on continues usage (β = 0.10, *p* = 0.14), recommendation (β = 0.02, *p* = 0.74), and WPP (β = 0.00, *p* = 0.91). Our results confirmed that age, gender, income are negatively correlated with continued use, recommendation, and willingness to pay premium. However, gender positively correlates with recommendation.

## Discussion and Contributions

### General Findings

Based on the TAM and theory of affective responses, this study builds up a pilot theoretical framework that depicts the key drivers of customer loyalty to VR-enabled services. In addition to the TAM, we verified that authenticity experience plays a determinative role in forming customer loyalty via affective responses such as enjoyment, EI, and flow state, indicating that authentic experience can become a critical antecedent to customer loyalty. This finding shows that when authentic VR contents are intrinsically rewarding and enjoyable, they become emotionally involved, and their flow state becomes well developed.

Affective responses have been extensively investigated in different fields, such as the pleasure state of emotions evoked by the store environment (Brengman et al., 2012). Virtual atmospheric cues influence emotions and emotionally laden customers have an impact on consumer behavior (Loureiro and Ribeiro, 2014). Previous studies extensively investigate the impact of ease of use, usefulness, and enjoyment on behavioral intentions (Huang et al., 2016; Li and Chen, 2019; Manis and Choi, 2019), but they ignore the important mechanism; the mediation effect of enjoyment, emotional involvement, and flow in the relationship of drivers and customer loyalty.

Affective responses, such as enjoyment, EI, and flow state, are mediators of the driving effects in most dimensions. For example, Kim et al. (2020a) argued that users are frequently involved in VR services as a novel technology for hedonic incentives (e.g., enjoyment, EI, and flow state), which is consistent with the findings of Davis (1989). Similarly, Huang et al. (2013) reveal that hedonic elements, such as enjoyment, EI, and flow state, are important predictors to consumer behavioral intentions in experiencing the 3D tourism sites. These findings are not aligned with the findings of Huang et al. (2017); they studied the impact of online game user experience on consumer behavior in terms of word of mouth through the mediation effect of emotions. Their study revealed that emotions (pleasure and arousal) did not mediate the relationship of online game user experience (functional experience and social experience) and consumer behaviors in terms of word of mouth.

Furthermore, this study verified that affective responses are significant mediators between drivers of VR and customer loyalty behavior to VR-enabled services.

VR consumers’ flow state is an antecedent to continued use, indicating that continued use of VR-enabled services is an outcome of flow state in VR and is an essential factor for the future behavior of VR users. To encourage recommendation behavior, three dimensions of affection, flow state, enjoyment, and EI show positive strength; this indicates that if users enjoy VR services and experience flow state and become emotionally involved in VR services, they are more likely to recommend VR services to their fellows.

The findings on WPP are similar to that of recommendation. All the three affective responses (EI, flow, and enjoyment) are significant triggers of WPP, indicating that consumers are willing to pay higher prices for VR-enabled services for aroused affection.

Together, the findings indicate that engaging users emotionally enhances users’ favorable feelings and immersive experience can contribute positively to the formation of customer loyalty to VR services.

However, out of our supposition, a few links are not verified. Ease of use has a non-significant influence on EI, indicating that VR services may be easy to use and do not influence EI in our study context. These findings contradict the study of Huang et al. (2017); their study revealed that functional experience positively and significantly affects emotions (pleasure and arousal). In turn, emotions also positively and significantly impact consumer behavior in terms of word of mouth. Maybe in MMORPG, players are more involved and mobilize to counter-attack to the enemy, which is the greatest reason to trigger the emotions of online game players. These emotions are less triggered in VR services as they are less involved in it as compared to MMORPG. Enjoyment suggested contradictory results with continued use of VR-enabled services. Possibly, users may not enjoy VR services due to motion sickness when they put on VR headsets. Such dizziness happens when the brain receives conflicting signals when the environment where the individual is standing is moving around, and the brain equilibrium is disturbed. An exciting result regarding EI of non-supported link with continued use of VR is that EI may indirectly influence continued use of VR services, and findings may vary in specific VR services.

Affective responses show non-significant mediation results between drivers and continued use of VR, suggesting that pleasurable experience, EI, and immersion experience may not be enough to motivate users to use VR services in the future. However, results in our study context may vary in other study contexts. Similarly, non-significant mediation results of EI were reported between ease of use and recommendation and WPP. The possible reason is that ease of use of VR has a strong direct effect on customer loyalty dimensions rather than through EI, indicating that ease of use of VR is an important element in customer loyalty behavior.

In addition, we recognized that only 2% of the participants were over 31 years old/mature people is a threat to the generalizability of the conclusions in other ages or contexts. So, we limit the scope of conclusions to the segment of youngsters. This is rational because of the fact that VR services are quite new in many countries, and the marketers mainly target users of 18–25 years old at the time being.

### Contributions

Our study results have notable implications for academics and practitioners. From an academic perspective, this study contributes to the knowledge of customer loyalty in the context of VR-enabled services. VR services have increasingly attracted the attention of researchers. Despite the considerable attention given to VR technology, research on the drivers of VR-enabled services that affect customer loyalty in the context of marketing is limited. However, researchers have addressed consumer response to VR technology adoption in the context of marketing (Loureiro et al., 2019; Martínez-Navarro et al., 2019; Wedel et al., 2020). This research integrates the TAM, authenticity experience of VR with affective responses, including flow, enjoyment, and EI, to understand customer loyalty to VR-enabled services.

This research verifies that the TAM model combined with authenticity experience have a significant effect on affective responses, thus contributing to the VR literature and extending previous studies on the relationship between authenticity, TAM, and emotions (Huang et al., 2013; Kim et al., 2020a). The strong connection of authentic experience and affective response in our research reveals that VR-enabled services are closely associated with the emotional and immersive experience and extend earlier studies’ results (Guttentag, 2010; Tussyadiah et al., 2018; Kim et al., 2020a). VR services provide users interface efficiency, influencing consumer emotions because of fun and pleasure, engaging them emotionally, and moving away from their daily activities. Thus, the study theoretically validates that the TAM and authenticity are essential factors in VR-enabled services.

This study also validates the mediating role of affective responses for developing customer loyalty in VR-enabled services. Our findings suggest that affective responses are the predictors of customer loyalty. Several authors examined that consumer affective responses are the critical hedonic construct to understand consumer VR consumption intention (Huang et al., 2013; Li and Chen, 2019; Kim et al., 2020a). Insufficient evidence can be found regarding customer loyalty in the VR context. For example, Huang et al. (2013) asserted that emotions are important indicators of behavioral intentions in VR tourism marketing. Kim et al. (2020a) discovered that affective responses significantly affect consumer intention to travel destinations shown in VR.

Additionally, this study reveals that affective responses significantly impact customer loyalty, thus extending the knowledge on the relationship between affective responses and customer loyalty; no study has addressed it so far. Therefore, this study can help researchers understand the effect of emotional responses in VR settings. For instance, when users feel fun, pleasure and get emotionally involved in VR-enabled services, they develop a flow state, contributing to customer loyalty (i.e., continued use, recommendation, and WPP).

This study is the first to investigate customer loyalty to VR-enabled services because previous studies were focused on adoption, for instance, VR technology adoption in the context of marketing (Loureiro et al., 2019; Martínez-Navarro et al., 2019; Wedel et al., 2020) and adoption intension or willingness to purchase VR services (Tavakoli and Mura, 2015; Loureiro et al., 2019; Kim et al., 2020a). This study further adds to VR literature by verifying drivers and affective responses are key predictors of customer loyalty to virtual reality-enabled services.

### Marketing Implications

The findings of our study suggest that managers should emphasize the functional aspect of VR services, including usefulness and easiness, if they want to immerse users completely in VR-enabled services. VR content should be beneficial, useful, and knowledgeable so that it can be promoted quickly through online platforms, such as websites, and different social media platforms. For example, VR practitioners can add sensory features of VR gratified with video, audio, and voice recognition, including AI, so that target customers could immerse emotionally and be entertained by interactive VR services.

Our study provides fruitful insights for VR practitioners and businesses. According to our findings, the authenticity of the VR experience has a strong influence on EI, flow state, and enjoyment, which influence consumer loyalty to VR-enabled services. In addition, managers who want to add VR services in their business should design their VR content to be unique, appealing, and genuine, enhancing the consumers’ perception of authenticity in VR-enabled services. Managers may advance the emotional factors of consumers with high-resolution graphics and enable users to touch virtually. Therefore, managers should create VR content that is enjoyable, fun, be pleasurable, and the content must be captivating enough to boost the emotional factors in VR-enabled services.

Researchers found that the flow state positively affects the continued use of VR services, suggesting that VR stakeholders and managers should attract consumers by offering an improved functional experience. EI, flow state, and enjoyment positively and significantly affect the recommendation during VR interaction. Managers should promote hedonic attributes in the design and production of VR content to influence consumers’ emotional responses. As a result, users can quickly receive psychological satisfaction in engaging in VR services to the next level. They will recommend the services to their colleagues and pay an extra price for services. Managers should consider these aspects of VR technology in their marketing strategy.

### Limitations and Future Recommendations

This research also has some limitations—first, this research utilized cross-sectional data, thus limiting generalizability across other segments. Longitudinal data are a better option for future research. Future studies are advised to use more objective data collection, for instance, observation methods. Furthermore, we recognized that only 2% of the participants were over 31 years old/mature people is a threat to the generalizability of the conclusions in other ages or contexts. We suggest scholars to investigate samples over 30 years old to generalize the findings of this study in a variety of countries in the future when VR service providers are targeting more mature people.

Second, we studied functional factors of VR and the authenticity of VR in understanding the outcomes of VR experience. Future studies can examine experiences apart from functional aspects and authentic experiences. Third, we could not measure cybersickness issues; future research should find ways to measure cybersickness with objective physiological measurements or subjective questionnaires. Lastly, this study only investigated the possible determinants of VR acceptance to continued use, recommend, and WPP, as well as effective responses. Future research should examine other influential factors such as cultural, social, or psychological factors.

## Data Availability Statement

The original contributions presented in the study are included in the article/supplementary material, further inquiries can be directed to the corresponding authors.

## Ethics Statement

Ethical review and approval was not required for the study on human participants in accordance with the local legislation and institutional requirements. The patients/participants provided their written informed consent to participate in this study.

## Author Contributions

IA and JY: conceptualization and methodology. IA and RA: software. IA: validation, formal analysis, investigation, resources, data curation, visualization, and writing-original draft preparation. IA and JY: writing-review and editing. YC: supervision. All authors have read and agreed to the published version of the manuscript.

## Conflict of Interest

The authors declare that the research was conducted in the absence of any commercial or financial relationships that could be construed as a potential conflict of interest.

## Publisher’s Note

All claims expressed in this article are solely those of the authors and do not necessarily represent those of their affiliated organizations, or those of the publisher, the editors and the reviewers. Any product that may be evaluated in this article, or claim that may be made by its manufacturer, is not guaranteed or endorsed by the publisher.

## Footnotes

1 VR entertainment/music/movie, VR shopping, VR showrooms, VR tourism, VR games/racing/roller coaster, VR skydiving, and VR real estate.
